# Supraglottic Localization of IgG4-Related Disease—Rare and Challenging Equity

**DOI:** 10.3390/jpm12081223

**Published:** 2022-07-27

**Authors:** Magda Barańska, Joanna Makowska, Małgorzata Wągrowska-Danilewicz, Wioletta Pietruszewska

**Affiliations:** 1 Department of Otolaryngology, Head and Neck Oncology, Medical University of Lodz, 90-419 Lodz, Poland; wioletta.pietruszewska@umed.lodz.pl; 2 Department of Rheumatology, Medical University of Lodz, 90-419 Lodz, Poland; joanna.makowska@umed.lodz.pl; 3 Department of Pathomorphology, Central Clinical Hospital, Medical University of Lodz, 90-419 Lodz, Poland; malgorzata.wagrowska-danilewicz@umed.lodz.pl

**Keywords:** IgG4-related disease, supraglottic stenosis, localized fibroinflammatory disease

## Abstract

Supraglottic stenosis is a rare symptom, particularly in fibroinflammatory multifocal diseases, such as IgG4-related disease (IgG4-RD). There is still an inconsistency in the diagnosis of less-common locations of IgG4-RD, which causes a delay in the diagnosis and treatment. Our paper aims to analyze different aspects of IgG4-RD presenting as supraglottic stenosis, including the possible overlap with ANCA-associated vasculitis. We compare the usefulness of the recently revised ACR/EULAR and Comprehensive criteria and discuss treatment options. The review was performed according to PRISMA guidelines using the MEDLINE Pubmed and Scopus databases. The analysis includes nine papers describing supraglottic laryngeal stenosis in 13 patients. Furthermore, we present a case of a woman with ongoing supraglottic stenosis presenting with cough, temporary dyspnea and stridor as the symptoms of localized IgG4-RD. At the time of writing, the patient remains in remission while receiving treatment with cyclophosphamide and methylprednisolone. The symptoms of supraglottic localization of IgG4-RD may be severe; however, at that point, clinicians should suspect autoimmune etiology and attempt to modulate the autoimmune response instead of performing dilatation surgery—the effects of which may not result in extended intervals between interventions. The ACR/EULAR criteria show great specificity; however, when IgG4-RD is presumed, the specific treatment should be implemented.

## 1. Introduction

IgG4-related disease (IgG4-RD) is a relatively new fibroinflammatory clinical entity with a history dating back to 2003. Previously, the disease was referred to as autoimmune pancreatitis, retroperitoneal fibrosis, Kuttner tumors, Mikulicz’s disease, inflammatory pseudotumor, aortitis, Riedel thyroiditis and lymphadenopathy [1]. The disease may manifest in any location that results in a large spectrum of involved organs: the thyroid, kidneys, prostate, skin, lungs and others [2,3,4]. The disease is often described as subacute with varied symptoms depending on the organ affected. When IgG4-RD is present in vital organs, this poses a need for immediate and thorough treatment [1,2]. 

In the head and neck region, it typically causes adenopathy, inflammatory pseudotumor or sialadenitis [5]. The lesions are characterized by dense lymphoplasmatic infiltrate with IgG4-positive plasma cells and storiform fibrosis. Some patients have elevated serum IgG4 levels [2]. The etiology of the disease is not fully understood; however, the genetic background and role of bacteria, i.e., *Helicobacter pylori* have been discussed [2]. The disease typically affects middle-aged and older people—90% of patients with IgG4-RD are 50–80 years old [1]. The disease often presents as multiorgan; however, IgG4-RD may present as an isolated tumorous swelling [2].

Among a large spectrum of symptoms, supraglottic stenosis is an extremely rare finding in patients diagnosed with IgG4-RD, particularly when it appears as an isolated disease manifestation. Injury of the semi-rigid structure of larynx and healing process leads to formation of concentric scars and thereafter progressive narrowing and airway compromise [6]. In the literature, we found only 13 cases of the disease localized in the region [7,8,9,10,11,12,13,14,15]. Patients usually complained of shortness of breath, dysphagia, dysphonia or hoarseness and, for that reason, were referred to ENT doctors.

The repeating pattern in these patients is numerous surgeries that they underwent including balloon dilatations, laser excisions and laryngotracheal reconstructions (Table 1). The questions addressed in the review were: “Is treatment of localized IgG4-related disease in the supraglottic region different from the IgG4-RD treatment of systemic disease?” and “What diagnostic pathway was taken in cases of the localized form of IgG4-RD in supraglottis?”

This article aims to review the available related literature and compare different approaches to the supraglottic localization of IgG4-RD. In the article, we review the available literature to describe and compare the incidence, clinical manifestation, treatment and outcomes in the supraglottic manifestation of IgG4-RD. Furthermore, our additional goal is to present a case of IgG4-RD causing isolated supraglottic stenosis.

## 2. Materials and Methods

**Figure 1:**
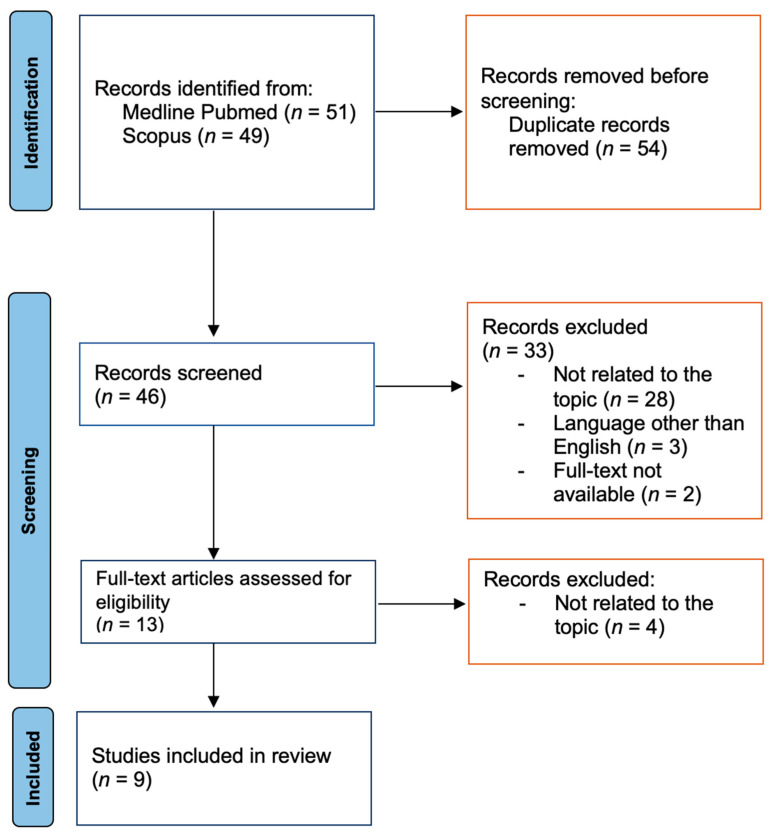
Study selection flowchart [18].

A comprehensive, PRISMA-compliant review was performed using MEDLINE PubMed and Elsevier SCOPUS database in April 2022 (Figure 1). We searched for articles with the following entries: “IgG4-related disease AND larynx”, “laryngeal immunoglobulin G4-related disease” and “IgG4-related disease AND supraglottic”. We searched for original articles, case reports and reviews. Articles with languages other than English were excluded.

Two investigators (WP and MB) independently screened the results. We included case reports wherein the patients were finally diagnosed with IgG4-RD based on histopathological and clinical criteria. From the cases, we selected those that described patients with supraglottic laryngeal stenosis confirmed in flexible nasolaryngoscopy. A total of 13 unique patients with supraglottic laryngeal stenosis in IgG4-RD have been reported in the literature. Furthermore, we attempted to classify the literature cases according to the Comprehensive and EULAR criteria (Table 1) [16,17].

## 3. Results

We identified a total number of 100 articles. After careful consideration, nine of them were included in our analysis (Figure 1). Table 1 presents the characteristics of 13 patients described in the nine above-mentioned studies and the case of a patient diagnosed and treated at the Department of Otolaryngology, Head and Neck Oncology, Medical University of Lodz, Poland. Our paper describes a case of a patient based on medical records; therefore, informed consent from the patient was not necessary to obtain.

### 3.1. Overview of Previously Published Case Reports

The chosen studies describe eight male and five female patients. One study included a child without defined age. In the adults’ group, the youngest patient was 29 years old, and the oldest was 76 years old. Female patients were reported to be more inclined to head and neck manifestations of the disease, which was not confirmed in the analyzed group of supraglottic stenosis [19]. The most frequently reported symptom by patients was dysphonia (10 patients), followed by dysphagia (seven patients) and then dyspnea (four patients), odynophagia stridor and cough (three patients). Other less-frequently observed symptoms included throat discomfort, general malaise, snoring and globus symptoms.

Several co-morbidities were described, including Sjogren*’*s syndrome, vasculitis, sclerosis cholangitis, rheumatoid arthritis, hypothyroidism and Helicobacter pylori infection, which cover the abovementioned IgG4-RD triggering factors. All the subjects were examined with flexible nasolaryngoscopy, which typically confirmed supraglottic and interarytenoid fibrosis, ulcer or diffuse swelling of the region, cicatricial narrowing and, in some cases, limited vocal fold mobility. Laser excision was performed in five cases; however, it had to be followed by another surgery. It was the pharmacological treatment that finally gave resolution of symptoms. The non-surgical approach included a wide range of medications: glucocorticoids, immunomodulatory drugs and cytostatic drugs; however, each of them is recommended in systhemic IgG4-RD [7,8,9,10,11,12,13,14,15,20].

### 3.2. Case Report of L.G.

A 72-year-old female patient L.G. presented with temporary dyspnea at rest, cough and globus symptoms. She had a 15-year history of hypothyroidism and glaucoma. No other significant diseases in her past or in family were described.

**Figure 2:**
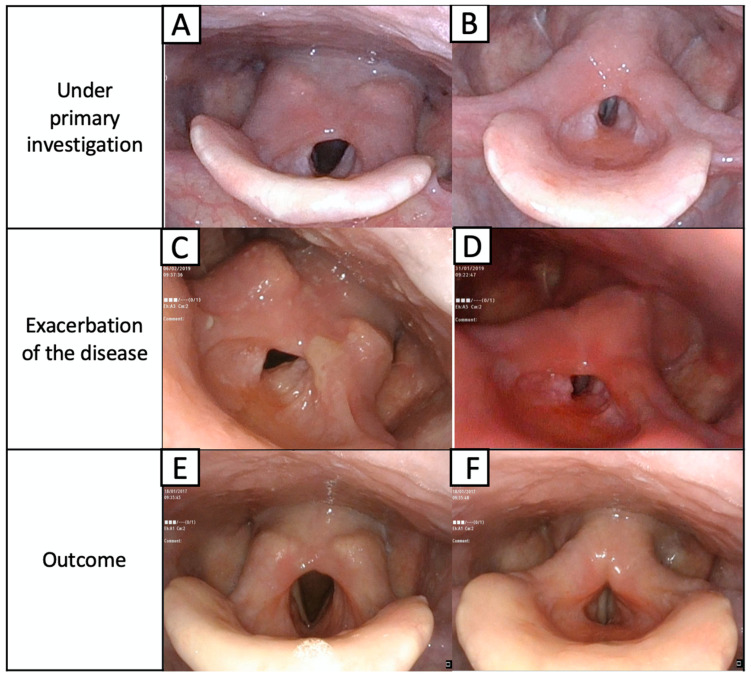
Laryngeal concentric stenosis of epiglottic area is mostly seen as an overgrowth of both the aryepiglottic folds and the posterior part of the larynx above the unchanged vocal folds. Mobility of the vocal folds is slightly limited by the supraglottic narrowing, which significantly reduces the larynx patency. Vocal folds are seen in the respiratory (**A**) and phonation phases (**B**). During the exacerbation of the disease, massive swelling of the supraglottic region and the progress of stenosis (**C**,**D**) are visible. The condition after 6 months of treatment from worsening of the disease, visible regression of the disease in the larynx during respiratory (**E**) and phonation phases (**F**).

At presentation at the Department of Otolaryngology, Head and Neck Oncology at the Medical University of Lodz, L.G. had severe supraglottic stenosis revealed in flexible nasolaryngoscopy. The laryngeal inlet was narrowed, and we observed thickening of the aryepiglottic folds, hypertrophy of posterior commissure with a tissue overhanging to the glottis and limited mobility of the vocal folds (Figure 2).

We excluded cancer, injuries, iatrogenic causes, bullous disease, pemphigus vulgaris and other common causes of stenosis. Biopsy taken in local anesthesia from the oral cavity mucosa did not reveal either IgG immunoglobulin deposits or C3 complements. However, a biopsy taken from the laryngeal lesion revealed nonspecific active chronic inflammation. A direct immunofluorescence test, antibodies on the substrate of stratum spinosum and transitional epithelium were unrevealing, opposite to positive cANCA antibodies (1:40) (PR3 ++) and positive SES ANA (1:640) with the parepidermoidal normotypic epithelium. This led to a provisional diagnosis of a local form of ANCA-associated vasculitis (granulomatosis with polyangiitis).

The patient’s condition initially improved after the methotrexate (20 mg/week) and prednisone (5 mg/day—starting from glucocorticoids pulses 3 × 1000 mg methylprednisolone and then 30 mg prednisone in descending doses). Full mobility of the larynx was restored. Due to poor tolerance and severe gastric complaints after 2 months of treatment, the methotrexate was changed to 150 mg azathioprine daily.

Despite the initial improvement, the dyspnea gradually worsened over the next several months. The patient was re-admitted to the hospital with stridor and dyspnea. Endoscopic examination revealed stenosis of the upper airway with edematous posterior commissure of the larynx, which immobilized the arytenoid cartilages and impaired the vocal fold mobility (Figure 2C,D). The pemphigus and pemphigoid were again excluded from consideration, and the IGRA tests for tuberculosis and cANCA antibodies were negative. A biopsy from the laryngeal lesion was taken under local anesthesia because the patient did not give consent to a tracheotomy. 

**Figure 3:**
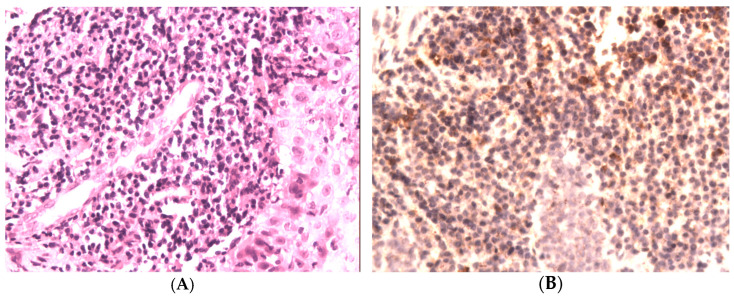
(**A**) Massive, subepithelial, mononuclear inflammatory infiltrates; hematoxylin and eosin. (H + E), magn. 200×. (**B**) Immunohistochemical staining for IgG4. The immunoexpression of IgG4 was present in most of the mononuclear cells of inflammatory infiltration (>50%), magn. 200×.

The histopathological examination of the sections from the supraglottic area revealed massive, subepithelial, mononuclear inflammatory infiltration (Figure 3A). Also, loose shreds of tissue with fibrosis were present. Then, immunohistochemical examination was performed (Zytomed, clone ZSIGG4, using the standard method). The immunoexpression of IgG4 was present in 50% of mononuclear cells of inflammatory infiltration (Figure 3B). Then, we assessed her serum IgG4 level, which was >135 mg/dL.

A definite diagnosis of localized IgG4-related disease was made [21]. Multifocal localization was excluded by CT examination of the chest and abdominal ultrasound. Immunosuppressive therapy with cyclophosphamide in intravenous pulses (1 g for every 4 weeks) and methylprednisolone (500 mg in pulses for 3 days and then 1 pulse for a month) was implemented. The patient remained in observation for over 12 months with significant improvement (Figure 2E,F).

## 4. Discussion

### 4.1. Diagnosis of IgG4-Related Disease: Comprehensive vs. ACR/EULAR Criteria

**Figure 4:**
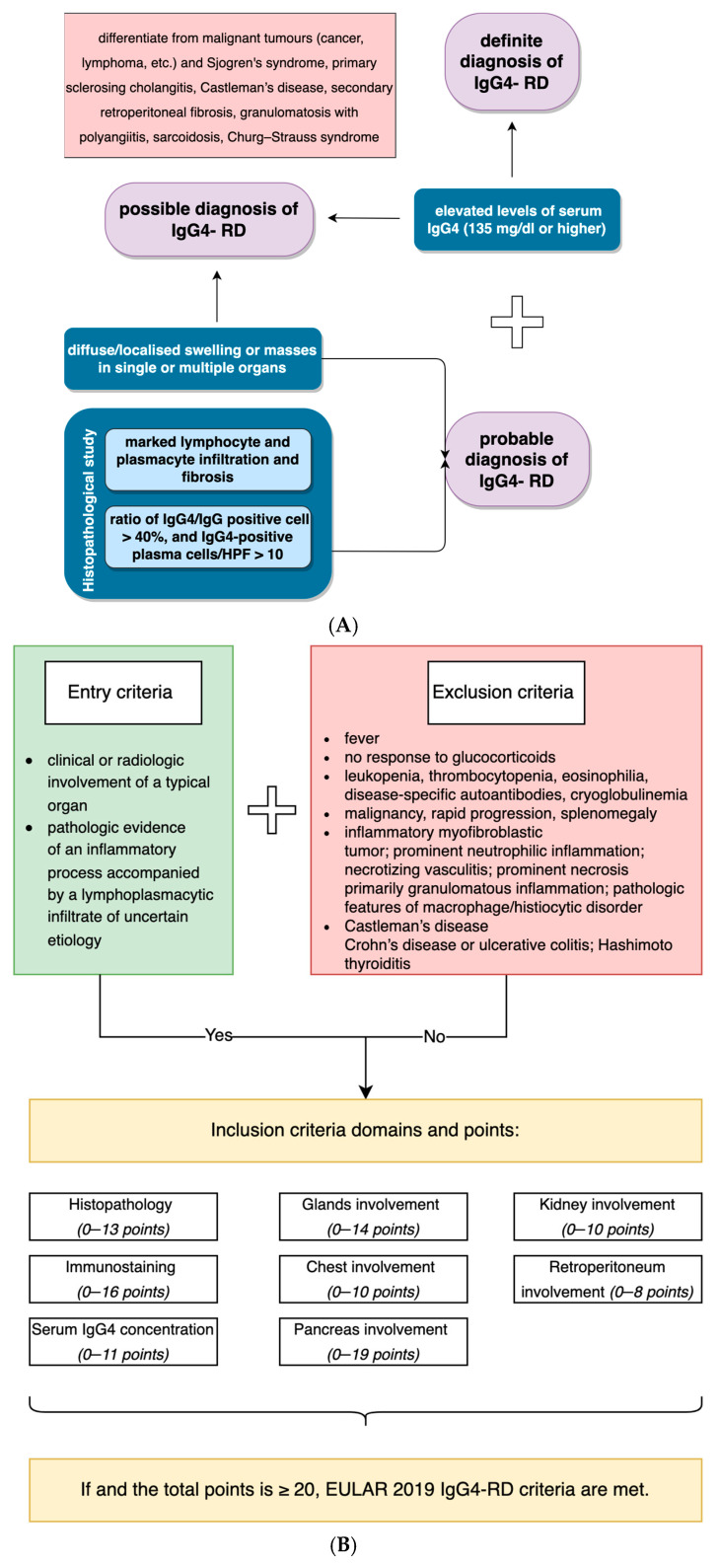
Comparison of the Comprehensive (**A**) and ACR/EULAR criteria for IgG4-related disease (**B**).

Diagnosis of IgG4-RD may be ambiguous, particularly when a non-specific and not common organ is affected. Currently, specific diagnostic criteria for laryngeal localization of IgG4-RD do not exist, as clinical trials are impossible due to the rare incidence and the fact that contemporary knowledge is typically based on case reports. We analyzed each case included in the study in terms of the Comprehensive and new ACR/EULAR criteria. Figure 4 briefly summarizes the main points of those [16,17].

Of 13 patients reported in the literature and patient L.G., nine had a probable diagnosis of IgG4-RD, and only four were definite. Some of those “probable” diagnoses were caused by an absence of IgG4 serum level, which may be either at a normal level or not assessed. On the other hand, it may disrupt an accurate diagnosis, as IgG4 serum levels are not routinely performed in hospitals, and its concentration may be elevated in numerous diseases [22].

Nine patients with “definite” or “probable” IgG4-RD fulfilled the ACR/EULAR criteria for IgG4-RD diagnosis and gained 20 or more points (Table 1). A similar comparison was made by Peters et al., who applied both consensus and 2019 ACR/EULAR criteria [23]. Of 68 patients with definite or probable diagnosis in criteria from 2021, 69% met the EULAR criteria for IgG4-RD [23]. The 2019 ACR/EULAR criteria showed great specificity; however, when probable diagnosis was made and specific treatment was implemented, patients benefited from long-time remission. It appeared that the new EULAR criteria have higher specificity in excluding the disease and, therefore, may prevent clinicians from over-diagnosing the disease, which has several mimickers.

### 4.2. Differential Diagnosis between IgG4-RD and ANCA-Associated Diseases

IgG4-RD is equally under- and over-diagnosed, as reported by Cheuk. Under-diagnosis results from low awareness of the disease, while over-diagnosis may be an effect of the increased level of IgG4+ cells [24].

The disease is likely to form tumefactive lesions, and, in the head and neck region, it may be misdiagnosed as ANCA-associated vasculitis or malignancy. Furthermore, the authors hypothesize that IgG4-RD and ANCA-associated vasculitis in rare cases may overlap, particularly owing to the fact that they share a clinical and histopathological features [25,26]. The ANCA-associated diseases include granulomatosis with polyangiitis, eosinophilic granulomatosis with polyangiitis (Churg–Strauss syndrome) and microscopic polyangiitis.

The distinction between IgG-RD and ANCA-associated vasculitis is crucial because these diseases have different prognoses and slightly different treatment approaches. This indicates the necessity to follow ACR and Chapel Hill for ANCA-associated vasculitis as well as IgG4-RD criteria to properly diagnose the diseases [27,28].

GPA affects patients at any age, with predominance in the sixth and seventh decade of life and can be localized in the larynx and trachea region; however, it often coexists with glomerulonephritis. In diagnosis, the presence of ANCA antibodies and histological examination of the fibrosis are necessary. A detailed scheme was suggested by the American College of Rheumatology [29]. Mild forms of GPA, with a prevalence 4–22%, may be localized in one organ [30]. However, in laryngeal manifestation, subglottic stenosis is the most common localization of narrowing [30]. The authors suggested that, in localized disease, particularly at its early stages, almost half of the patients may be ANCA-negative. Hence, when ANCA is elevated in the differential diagnosis, practitioners should focus on eosinophilic granulomatosis with polyangiitis [31].

The literature on the subject reports one case of a pediatric patient with a laryngeal lesion and finally diagnosed with Churg–Strauss syndrome. The lesion involved both vocal folds and the interarytenoid area. The mass was surgically removed but recurred after several months [32]. Nonetheless, the ACR criteria state that patient should also have asthma, a high eosinophilic level, nerve damage, migratory spots, sinus problems or white blood cells in the lesion, which was not fulfilled in our patient [33]. On the other hand, the authors described that vocal fold involvement may be associated with microscopic polyangiitis (MPA) (clinically, ENT involvement is observed in 2–20% of patients) [34]. However, ENT manifestation is officially not considered as an MPA symptom [35].

A study from 2012 examined the prevalence of IgG4+ cells in biopsies from GPA patients with manifestation in the head and neck region. They found that 8 out of 26 probes revealed an increased number of IgG4+ cells. Hence, granulomatosis with polyangiitis may be misdiagnosed as the inflammatory background, fibrosis and high proportion of IgG4+ plasma cells is present in both diseases. 

Another study described that the IgG4-positive cells may be present in 38% of GPA cases in the head and neck region [36]. A case of a pediatric patient with orbital swelling had about 50% IgG4-positive cells and was finally diagnosed as granulomatosis with polyangiitis [37].

In the case of L.G., the diagnosis of GPA with increased IgG4+ cells could have been made unless the criteria described in Figure 4 were met (particularly both the serum concentration and IgG4/IgG cell ratio). The cutoff value of 135 mg/dL was first defined in 2001, as 95% of patients with autoimmune pancreatitis had at least that IgG4 concentration in serum [38]. However, the IgG4 serum level is not necessary for the diagnosis [39]. However, the presence of granulomas exclude IgG4-RD from consideration [40].

Treatment varies in those two entities. In ANCA-associated vasculitis, the treatment is standardized leading to a low relapse rate. This treatment includes cyclophosphamide or rituximab with glucocorticoids [41]. On the contrary, treatment of IgG4-RD should be started with glucocorticoids, without immunosuppressive treatment in the first line [2,41]. Recent reports suggest that rituximab, anty-CD20 therapy or cyclophosphamide could be used in ANCA-associated vasculitis/IgG4-RD overlap syndrome [26,42,43].

### 4.3. Management of Supraglottic Stenosis in ENT Practice

**Figure 5:**
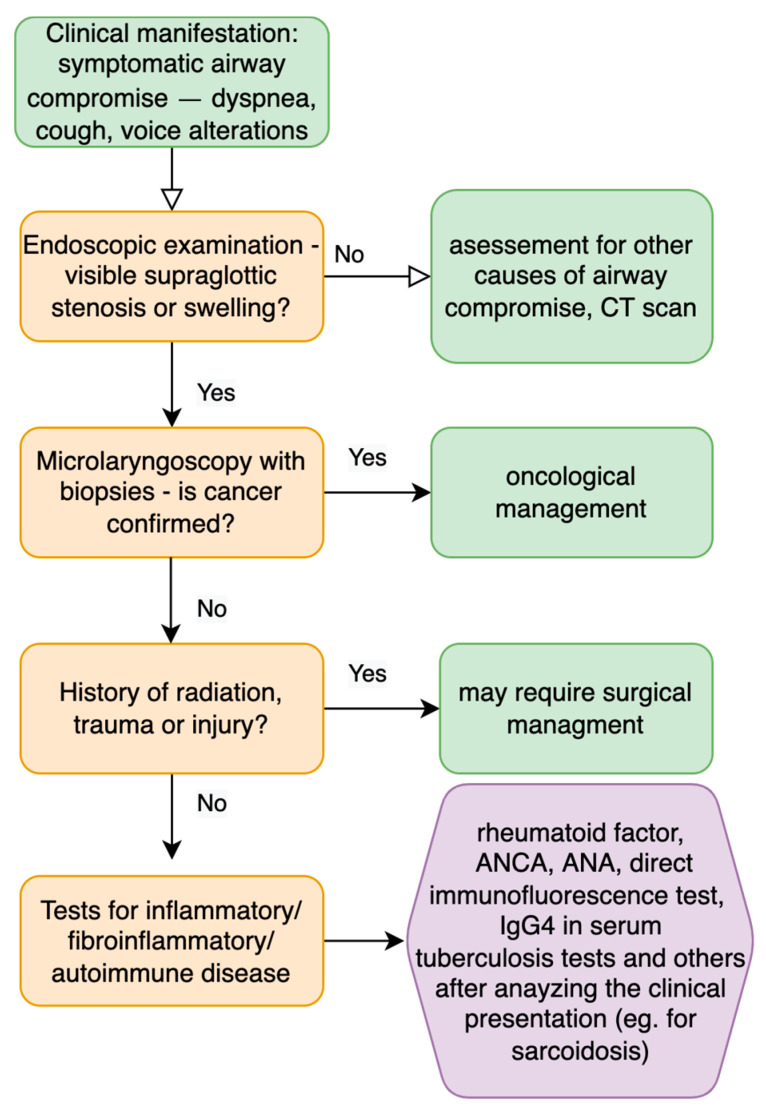
Diagnostic steps for patients with supraglottic stenosis and compromised symptomatic airway [6,48].

Supraglottic laryngeal stenosis stands for 3% of all laryngeal narrowing cases; however, due to the rare incidence, some authors do not confirm the prevalence numbers [44]. The most common causes include blunt or penetrating trauma, iatrogenic causes (after oncological treatment or wrong instrumentation), caustic or thermal burns, prolonged intubation [45], external beam irradiation, erosive lichen planus, pemphigoid, sarcoidosis and other autoimmune diseases [46,47]. Figure 5 presents a possible workup for a patient with supraglottic laryngeal swelling.

Treatment typically includes endoscopic surgery, open surgery or a combination of both. In severe cases, and if the final treatment is delayed, patients may need a tracheostomy [6,44]. As the nature of the condition is associated with recurrences, this reduces the quality of a patient’s life considerably and requires treatment modification from ENT doctors to ensure the longest possible period without increasing narrowing.

It is currently discussed in the literature whether the surgical approach used as the first line of treatment in stenosis of unknown origin is best for the patient. Some authors underline the necessity for immunological system modulation rather than mechanical treatment [49]. In the described case reports, five patients underwent surgery; however, for only one of them was it a long-term solution. Maughan described a patient who underwent laryngotracheal reconstruction with a good outcome. 

However, an open procedure significantly increases the risk, particularly when patients suffer from other co-morbidities compared to endoscopic procedures. On the other hand, the goal of extending the interval between interventions was achieved [7]. In the case of our patient, we started with a pharmacological approach, and we observed improvement without the need for surgery. We based our experience on previous cases of epiglottal stenosis, particularly granulomatosis with polyangiitis (GPA), in which dilatation stimulated rapid growth of the stenosis requiring tracheotomy.

**Figure 6:**
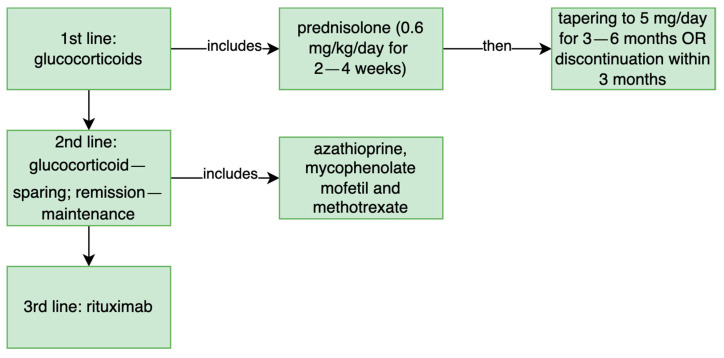
The treatment recommended in IgG4-related disease [1].

Other patients were finally treated with prednisolone, rituximab, azathioprine or methotrexate, which covers the recommended treatment in IgG4-RD. (Figure 6) The detailed report of the treatment applied in selected patients is described in Appendix A. Unfortunately, atypical manifestations respond poorly to conventional therapies [25].

## 5. Conclusions

Patients presenting with supraglottic laryngeal stenosis should have biopsies performed whenever possible and have those examined in search of IgG4-RD features. An appropriate workup can lead to a higher ratio of decannulation and reduce the voice and swallowing compromise. Furthermore, this may lead to a decrease in patient exposure to unnecessary surgeries. Laryngeal swelling, dyspnea and hoarseness may be the first isolated symptoms of IgG4-RD.

Clinicians should mind both the Comprehensive and 2019 ACR/EULAR criteria, as the first encourages a reduction in unnecessary surgical procedures in favor of the pharmacological approach and the latter reduces false-positive diagnoses, as IgG4 may easily resemble several autoimmune diseases. The goal for patients with supraglottic stenosis in ongoing IgG4-RD is to extend the period without intervention, and it appears that this may be achieved by immunomodulatory drugs.

## Figures and Tables

**Table 1:** Overview of selected studies. Abbreviations: F—female and M—male; Comprehensive criteria diagnosis: D—definite diagnosis and P—probable diagnosis; SL—IgG4 serum level; HPF—high power field; PPI—proton pump inhibitor; GI—gastrointestinal; and COPD—Chronic obstructive pulmonary disease.

Study	Sex, Age	Symptoms	Co-Morbidities	Disease Location	Endoscopic Examination	Treatment/Intervention	Outcome	IgG4 Serum Level and Histopathology	Comprehensive Criteria	EULAR Criteria
**Patient L.G.—current case**	F, 72	dyspnea at rest, cough, globus symptoms	hypothyroidism, glaucoma	supraglottic region	laryngeal inlet narrowing; hypertrophy; limited mobility of vocal folds	(1) methotrexate changed to azathioprine; prednisone; (2) cyclophosphamide; methylprednisolone	(1) poor toleration of methotrexate; stenosis progressed; (2) significant improvement	SL > 135 mg/dL; IgG4 in 50% of mononuclear cells	D	15
**Matsushima (2019)** [8]	M, 50	dyspnea; snoring	cerebral infarction, retroperitoneal fibrosis	left arytenoid region	diffuse swelling	(1) CO2 laser resection and tracheostomy; (2) prednisolone	1 and 2: improvement	SL 31 mg/dL; storiform fibrosis; >100 IgG4+ plasma cells; 50% IgG4:IgG	P	≥20
**Maughan (2020) patient 1:** [7]	F, 52	dyspnea; biphasic stridor	not reported	supraglottic, region	visible fibrosis	(1) balloon dilatation, excision, steroids; (2) prednisolone, azathioprine changed to methotrexate; (3) laryngotracheal reconstruction	transient relief after 1 and 2; (3) improvement	SL—normal; lymphoplasmocytic infiltrate, fibrosis, IgG4:IgG 80%	P	≥20
**Maughan (2020) patient 2:** [7]	M, 76	dysphagia, dysphonia	asbestos exposure, hypothyroidism, gastritis, H. pylori infection	supraglottic region	swelling	(1) balloon dilatation, laser excision, steroids; (2) immunomodulatory treatment	(1) repeated every 4–6 months (2) improvement	SL—normal; lymphoid infiltrate, plasma cells, 20% IgG4+ cells (50 IgG+ in HPF);	P	4
**Maughan (2020) patient 3:** [7]	M, 49	dysphonia; inspratory stridor	GI reflux, allergic rhinitis	supraglottic region	scarring, restricted arytenoid movement bilaterally	(1) balloon dilatation, laser excision, steroid injections; (2) prednisolone	(1) repeated dilatations; (2) follow up not described	SL—0.9 range; inflammation, fibrosis, ANCA- negative;	P	13
**Hamadani (2016)** [9]	F, 54	dysphagia, odynophagia, weight loss; dysphonia;	rheumatoid arthritis, liver cirrhosis, portal hypertension,	supraglottic, postcricoid region	visible mucus, postcricoid ulcer, laryngospasm	not described	not described	IgG lymphocytoid plasma-cell infiltrates, >90% IgG+ plasma cells	P	8
**Ferrante (2017)** [10]	F, 70	stridor, dyspnea at rest, dysphonia, dysphagia	Sjögren’s syndrome, rheumatoid arthritis, Felty syndrome, COPD	supraglottic region; nasopharynx;	anterior septal perforation, nasal wall scarring, supraglottic stenosis	tracheostomy; prednisolone	slow improvement; decannulation after 16 months	40 IgG4 plasma cells HPF, storiform fibrosis, lymphoplasmatic mucositis	P	≥20
Reder **(2015) patient 1:** [11]	M, 58	throat discomfort, dysphonia	semicircular canal dehiscence	supraglottis, right vocal process, aryepiglottic fold	visible lesions	(1) laser excision; (2) prednisolone; (3) rituximab, methylprednisolone	(1) no long-term improvement; (2) poor toleration; (3) remission	Sl—196 mg/dL; storiform fibrosis, lymphoplasmacytic infiltrate; 50 IgG4+ cells per HPF; IgG4:IgG > 0.50;	D	≥20
**Reder (2015) patient 2:** [11]	M, 62	cough, dysphagia, dysphonia	primary scleros- ing cholangitis, ulcerative colitis, and colorectal cancer	supraglottic region	granular mucosa, keratosis, hyperplasia	(1) prednisone; (2) rituximab, methylprednisolone	(1) “modest” clinical improvement; (2) significant improvement	SL—28.6 mg/dL; lymphoplasmacytic infiltrate and fibrosis; >100 IgG4+ cells;	P	≥20
**Reder (2015) patient 3:** [11]	F, 50	throat discomfort	hypertension and GI reflux disease	supraglottic region	ulcerative lesion of the left pharyngeal wall	Rituximab, methylprednisolone	significant improvement; normalization of IgG4 serum concentration;	lymphoplasmacytic infiltrate, storiform fibrosis; >50 IgG4+ plasma cells;	P	≥20
**Khoo (2014)** [12]	M, 62	cough, dysphagia, odynophagia, dysphonia, otalgia,	not reported	supraglottic region, aryepiglottic folds	supraglottic papilli- tumor involving the aryepiglottic folds bilaterally	prednisolone	significant improvement visualized in flexible laryngoscopy at 6 and 12 weeks	SL—154 mg/dL; plasmacytoid infiltrate; >50 IgG4+ cells per HPF; IgG4:IgG > 40%,	D	≥20
**Jordan (2018)** [15]	F, pediatric patient	dysphonia, globus symptoms, dysphagia	patient without comorbidities	epiglottis, arytenoids	thickening of tissues	rituximab, high-dose steroids	stabilization of disease for 18 months; reduction of laryngeal findings	increased number of IgG4+ cells with IgG:IgG4 40% to 50%	P	7
**Syed (2020)** [14]	M, 69	cough, dysphonia and dyspnea	multivessel coronary artery disease, lacunar cerebrovascular accident, hypertension, hyperlipidemia, benign prostatic hyperplasia	lacrimal gland, pancreas, epiglottis, vocal cord	epiglottic inflammation, vocal cord dysfunction,	(1) azithromycin, albuterol, histamine-2 receptor antagonist, PPI; (2) rituximab	(1) no effects; (2) improvement	SL—29 mg/dL; previous IgG4-RD diagnosis	D	≥20
**Hill (2020)** [13]	M, 29	odynophagia, dysphonia, dysphagia	reactive airway disease	arytenoid, aryepiglottic fold	limited mobility of the vocal fold	(1) doxycycline; (2) prednisone	(1) no effects; (2) improvement	Sl—133.6 mg/dL, inflammatory infiltrate, increased plasma cell component	D	≥20

## Data Availability

Not applicable.

## Appendix A

**Table A1:** Detailed diagnosis, treatment and outcome of the reviewed cases.

Study	Endoscopic Examination	Treatment/Intervention	Outcome	IgG4 Serum Level and Histopathology	Comprehensive Criteria	EULAR Criteria
**Patient L.G.—current case**	narrowing of laryngeal inlet; aryepiglottic folds thickening, hypertrophy of posterior commissure; limited mobility of vocal folds	(1) methotrexate (20 mg/week), changed to 150 mg azathioprine daily; prednisone (5 mg/day—starting from glucocorticoids pulses 3 × 1000 mg methylprednisolone, then 30 mg prednisone in descending doses). (2) cyclophosphamide (1 g for every 4 weeks); methylprednisolone (500 mg in pulses for 3 days and then 1 pulse for a month)	(1) poor toleration of methotrexate; after initial improvement stenosis increased; (2) significant improvement	IgG4 serum level >135 mg/dL; IgG4 in 50% of mononuclear cells of massive inflammatory infiltration from the biopsy specimens	definite diagnosis	15
**Matsushima (2019)** [8]	diffuse swelling—left arytenoid region, obscuring visualization of the glottis	(1) wide resection with CO2 laser; (2) tracheostomy (3) prednisolone (0.6 mg/kg/day); now 5 mg/day	reduction of tumor size after 2 weeks of treatment	serum IgG4 31 mg/dL; storiform fibrosis; >100 IgG4-positive plasma cells and 50% IgG4/IgG	probable diagnosis	≥20
**Maughan (2020) patient 1:** [7]	supraglottic and interarytenoid fibrosis	(1) balloon dilatation and microlaryngoscopy + laser excision + steroid injections; (2) oral prednisolone + azathioprine then switched for methotrexate; (3) laryngotracheal reconstruction	transient relief after 1 and 2 treatment; significant long-term improvement after treatment 3, back to oral intake and work	IgG4 serum level—normal; lymphoplasmocytic infiltrate, fibrosis, IgG4:IgG ratio 80% in biopsy specimens;	probable diagnosis	≥20
**Maughan (2020) patient 2:** [7]	supraglottic swelling	(1) balloon dilatation and microlaryngoscopy + laser excision + steroid injections; (2) immunomodulatory treatment then for 30 months patient declined treatment	(1) had to be repeated every 4–6 months (2) patient for 30 months remains in “watch and wait” approach	IgG4 serum level normal; subepithelial lymphoid infiltrate, plasma cells, 20% positive cells for IgG4 (50 IgG+ in high power field);	probable diagnosis	4
**Maughan (2020) patient 3:** [7]	supraglottic scarring; restricted arytenoid movement bilaterally	balloon dilatation and microlaryngoscopy + laser excision + steroid injections	dilatation repeated every 4 to 6 months; after the diagnosis patient got the prednisolone; however, no further follow up is described	chronic inflammation, fibrosis, IgG serology 0.9 range; ANCA- negative; confirmed on biopsy specimens	probable diagnosis	13
**Hamadani (2016)** [9]	mucus in the supraglottis, postcricoid region ulcer, laryngospasm	treatment not described	outcome not described	IgG lymphocytoid plasma-cell infiltrates, with >90% of IgG-positive plasma cells that were IgG4-positive	probable diagnosis	8
**Ferrante (2017)** [10]	anterior septal perforation, lateral nasal wall scarring, supraglottis cicatricial narrowing down to 4 mm in diameter	tracheostomy; prednisolone 40 mg/d lowered to 10 mg/d	slow improvement; decannulation after 16 months	40 IgG4 plasma cells in high-powered field, storiform fibrosis, lymphoplasmatic mucositis	probable diagnosis	≥20
**Reder (2015) patient 1:** [11]	lesions on the base of tongue extending to aryepiglottic fold, right vocal process;	(1) laser excision of the lesion; (2) prednisolone 40 mg/day for 2 weeks; (3) rituximab 1 g—2 doses, 2 weeks apart, methylprednisolone 100 mg/day with every rituximab infusion	(1) second excision without long-term improvement; (2) poor toleration of prednisolone; (3) for 2 years patient remains in remission	IgG4 serum level: 196 mg/dL; polypoid squamous mucosa; diffuse storiform fibrosis, dense lymphoplasmacytic infiltrate; 50 IgG4-positive cells per high-power field; IgG4:IgG ratio > 0.50;	definite diagnosis	≥20
**Reder (2015) patient 2:** [11]	granular mucosa—base of the tongue and the epiglottis; keratosis, hyperplasia of the aryepiglottic folds, the false and true vocal cords	(1) 2 courses of prednisone 60 mg for 7 days, then 7-day taper; (2) rituximab 1 g—2 doses, 2 weeks apart, methylprednisolone 100 mg/day with every rituximab infusion	(1) “modest” clinical improvement; (2) significant improvement	serum level: 28.6 mg/dL; intense lymphoplasmacytic infiltrate and fibrosis; >100 IgG4-positive plasma cells;	probable diagnosis	≥20
**Reder (2015) patient 3:** [11]	ulcerative lesion of the left pharyngeal wall	rituximab 1 g—2 doses, 2 weeks apart, methylprednisolone 100 mg/day with every rituximab infusion	significant improvement; normalization of IgG4 serum concentration;	proliferative squamous mucosa with a lymphoplasmacytic infiltrate and storiform fibrosis; >50 IgG4-positive plasma cells;	probable diagnosis	≥20
**Khoo (2014)** [12]	supraglottic papilli- tumor involving the aryepiglottic folds bilaterally	37.5 mg prednisolone daily for 6 weeks, then 25 mg for 6 weeks with dose reductions to 5 mg	significant improvement visualized in flexible laryngoscopy in 6 and 12 weeks	serum IgG4 level: 154 mg/dL; dense plasmacytoid infiltrate in the subepithelial tissue with lymphocytes; significant staining with IgG4, in some areas with >50 stained cells per high-power field; IgG4:IgG > 40%,	definite diagnosis	≥20
**Jordan (2018)** [15]	surgically absent palatine tonsils, enlarged lingual tonsils, thickened epiglottis and arytenoids, fullness in the piriform sinuses, supraglottis thickening	high-dose steroids and rituximab	stabilization of disease for 18 months; reduction of laryngeal findings	increased number of IgG4- positive cells with IgG/IgG4 ratio of 40% to 50%	probable diagnosis	7
**Syed (2020)** [21]	gross inflammation of the epiglottis and vocal cord dysfunction,	(1) azithromycin albuterol inhaler, histamine-2 receptor antagonist, proton pump inhibitor (PPI) (2) rituximab	(1) no effects; (2) resolution of symptoms	IgG4 serum level 29 mg/dL; patient previously diagnosed with IgG4-RD; PET scan showed increased uptake in the larynx and thoracic aorta;	definite diagnosis	≥20
**Hill (2020)** [13]	right arytenoid extending into the aryepiglottic fold, limiting the mobility of the right vocal fold	(1) doxycycline; (2) prednisone orally	(1) no effects; (2) resolution of symptoms	IgG4 serum level 133.6 mg/dL, inflammatory infiltrate and an increased plasma cell component	definite diagnosis	≥20
